# Global longitudinal strain in the prediction of significant coronary artery disease: how accurate is it for patients with a high clinical probability of chronic coronary syndrome and preserved left ventricular ejection fraction?

**DOI:** 10.1186/s44156-025-00084-1

**Published:** 2025-07-01

**Authors:** Mame Madjiguene Ka, Serigne Cheikh Tidiane Ndao, Waly Niang Mboup, Mariama Barry, Rabab Yassine, Pape Momar Guissé, Demba Waré Baldé, Tacko Niang, Djibril Marie Ba, Khadidiatou Dia, El Hadji Mbacké Sarr, Ibrahima Bara Diop, Mouhamed Chérif Mboup

**Affiliations:** 1 Principal Hospital of Dakar, Dakar, Senegal; 2 Military Hospital of Ouakam, Dakar, Senegal; 3 National Hospital Center of Fann, Dakar, Senegal

**Keywords:** Global longitudinal strain, Chronic coronary syndrome, Diagnostic performance, SYNTAX score, Regional longitudinal strain, sub-Saharan Africa

## Abstract

**Background:**

GLS is a non-invasive imaging test that can be useful in the selection of patients highly suspected of CCS for coronary angiogram.

**Aims:**

This study aimed to evaluate the diagnostic performance of rest 2D speckle tracking echocardiography (2D-STE) for detecting obstructive coronary artery disease (CAD) in patients with high clinical probability of chronic coronary syndrome (CCS) and preserved left ventricular ejection fraction (LVEF).

**Methods:**

A prospective study enrolled 52 patients referred for coronary angiography due to highly suspected CCS. Participants were divided into CAD+ (significant stenosis) and CAD- (normal or non-significant stenosis). Transthoracic echocardiography (TTE), exercise EKG, 2D-STE, and coronary angiography were performed. Global longitudinal peak systolic strain (GLS) was calculated using 2D-STE, with a cut-off value of -18% for normal GLS. Reproducibility was assessed with intraclass correlation.

**Results:**

The mean age of participants was 62.5 ± 11.9 years, and 63.5% were male. The CAD + group (51.9%) had significantly higher rates of hypertension, diabetes, dyslipidemia, and typical angina. GLS was significantly lower in the CAD + group (-15.89 ± 2.07%) compared to the CAD- group (-18.99 ± 2.37%, *p* = 0.0001). The optimal GLS cut-off for detecting significant coronary lesions was − 16.9%, with 74% sensitivity, 76% specificity, and an area under the curve (AUC) of 0.83 (95% CI 0.73–0.94). GLS correlated with the number of diseased vessels (*p* = 0.0001) but not with lesion complexity (SYNTAX score, *p* = 0.18). Regional strain was significantly reduced in patients with obstructive lesions in the left anterior descending (LAD) and circumflex arteries (CX), with optimal cut-offs at -19.2% and − 15.8%, respectively. GLS showed excellent inter-operator reproducibility (ICC = 0.94, *p* < 0.0001).

**Conclusion:**

GLS demonstrates good diagnostic performance in detecting obstructive CAD in patients with a high pre-test probability of CCS and preserved LVEF. It serves as a reliable, reproducible indicator of significant coronary lesions, with promising clinical utility for non-invasive CAD assessment, particularly in resource-limited settings.

## Introduction

Cardiovascular diseases are the leading cause of mortality worldwide, especially in low and middle-income countries (LMIC), where 82% of deaths occur [1]. Coronary Artery Disease (CAD) is the most prevalent and fatal of them [2]. In Senegal, monocentric studies revealed that the hospital prevalence of acute coronary syndromes has been steadily increasing, from 4% in 2006 to 10% in 2022 [3].

Early detection and management of CAD are key challenges in cardiology. Recent European Society of Cardiology (ESC) guidelines recommend non-invasive imaging tests, such as coronary CT angiography or functional imaging, for symptomatic patients when obstructive CAD cannot be ruled out clinically, depending on availability, cost, and expertise [4]. The exercise ECG is widely available but has low sensitivity and specificity [5]. Nuclear imaging offers high accuracy but is limited by radiation, cost, and availability. Dobutamine stress echocardiography is cost-effective and radiation-free with high sensitivity and specificity, though the need for specialized expertise and the availability of intravenous beta-blockers in some restricted resource areas limit its use [4].

There is a shortage of these non-invasive diagnostic tools in developing countries or any resource-limited area such as Senegal. A simple, reliable, and widely available test is needed to enhance the prediction of severe coronary lesions and improve patient selection for coronary angiography.

The gradual adoption of strain echocardiography for diagnosing and risk-stratifying heart diseases has shown its value for patients suspected of CAD [6]. Indeed, 2D speckle tracking can evaluate typical subendocardial ischemic damage through multiple parameters: the global longitudinal strain (GLS) and the bull’s-eye representation of strain, which provides a regional assessment of ventricular segments based on coronary vascular territories. The latest ESC guidelines for diagnosing and managing chronic coronary syndrome (CCS) suggest the use of 2D speckle tracking with resting transthoracic echocardiography (TTE) to enhance diagnosis in patients suspected of CAD without kinetic abnormalities, owing to its higher diagnostic accuracy in detecting ventricular dysfunction despite preserved left ventricular ejection fraction (LVEF) [3].

Several studies worldwide have evaluated the contribution of 2D strain in diagnosing chronic coronary syndrome [7–11], but to our knowledge, none have been conducted in West Africa. In our context, widely adopting this tool (instead of exercise EKG) could save patients’ health expenditures and optimize the patients’ selection for further invasive tests.

## Objective

This study aimed to test the diagnostic performance of rest 2D speckle tracking for detecting obstructive CAD in patients with a high clinical probability of CCS and preserved LVEF.

## Materials and methods

### Study population and exclusion criteria

We performed a prospective study from October 1st, 2023, to June 30th, 2024, among patients referred to our cardiology department in the Principal Hospital of Dakar selected for coronary angiography due to suspected chronic coronary syndrome. These patients were:

- aged 18 + years.
- symptomatic with a high clinical probability of CCS: high score according to the 2019-ESC-PTP model [12], or intermediate score with at least one of the following conditions (diabetes, hypertension, dyslipidemia, tobacco smoking, resting ECG changes, abnormal exercise ECG test).
- No kinetic abnormalities were observed, and LVEF was preserved at resting echocardiography.

Patients with a documented previous ACS, ischemic heart disease, PCI, open heart surgery, severe valvular or myocardial disease, uncontrolled high blood pressure, Q waves at resting ECG, drug therapy that impairs cardiac function, chronic kidney disease, or presenting factors that challenge the interpretation of the 2D speckle tracking echocardiography (2D-STE) results (arrhythmia, conduction abnormalities, poor acoustic window) were excluded.

### Data collection

Transthoracic echocardiography (TTE), resting and exercise EKG, 2D-STE, and coronary angiography were performed.

TTE images were attained at rest using Vivid E9 (General Electric, USA) with an M4S transducer, with a 1.5–4.3 MHz frequency and a high frame rate (60–90 frames/s). We recorded three uninterrupted cardiac cycles for the three apical views (four-, two-, and three-chamber views). LV volumes were traced manually at end-diastole and end-systole, and LVEF was derived from the modified biplane Simpson method.

**Fig. 1:**
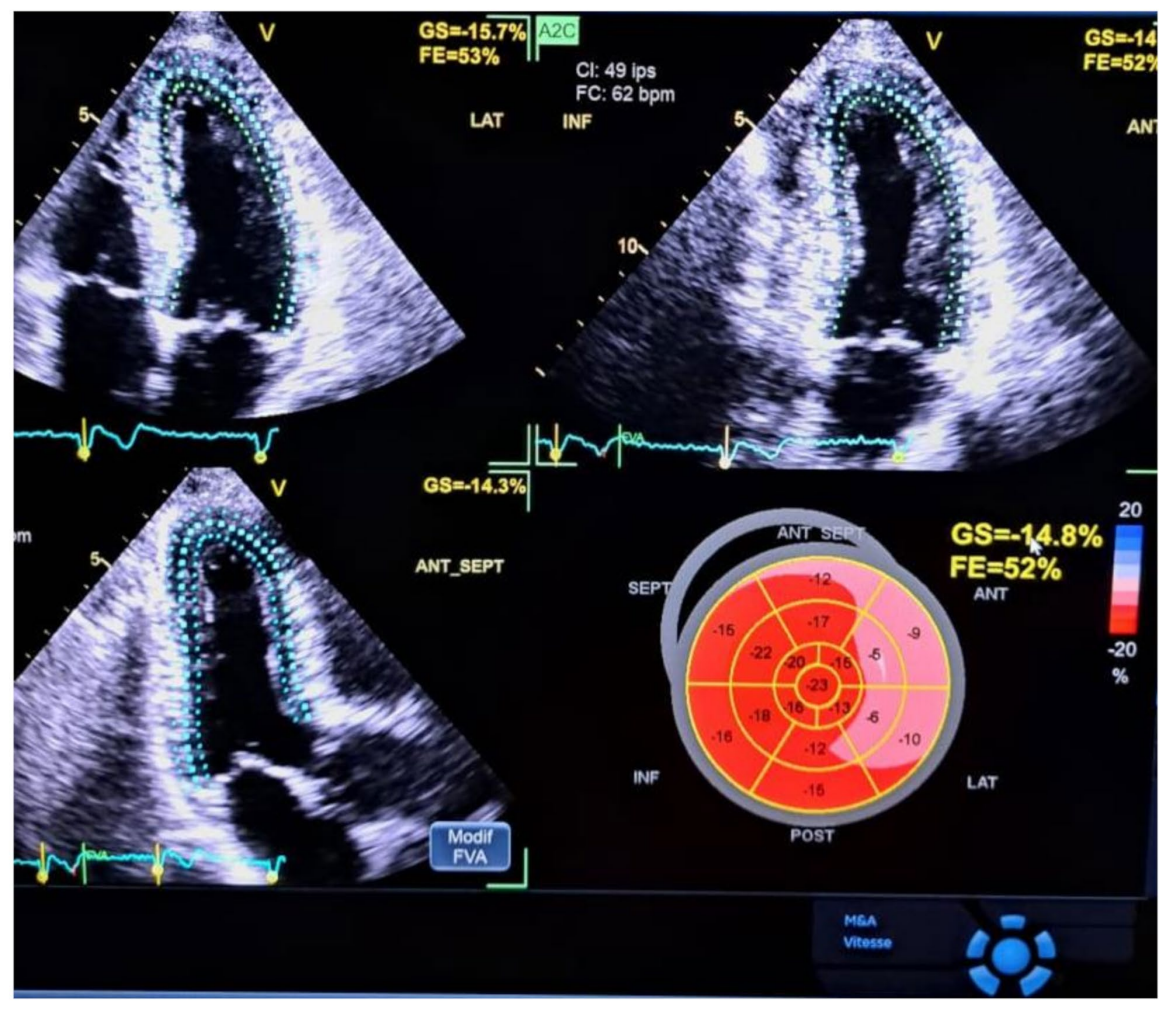
Global longitudinal strain measurement by 2D-STE with results in French (Principal Hospital of Dakar). Speckle tracking is shown in the three apical views. LVEF (FE in the figure) is normal, but GLS (GS in the figure) is impaired, with strain values at -14.8%. Regional longitudinal peak systolic strain (RLS) is markedly decreased in the CX territory

For the 2D-STE analysis, the endocardium was manually marked out from selected cine loops of all three apical view images. The software executed Visual confirmation of tracking after automatic deformation tracking. Segments with insufficient tracking were eliminated. Regional longitudinal peak systolic strain (RLS) was measured in all views from aortic valve opening to closing across six basal, six midventricular, and four apical segments, plus the apical cap (17th segment). These were averaged to calculate the global longitudinal peak systolic strain (GLS) (Fig. 1). The cut-off point for regular negative GLS was considered 18%.

To assess the inter-operator reproducibility, a second evaluation of 2D-STE was performed by an experienced echocardiographer on a 17-patient sample.

An interventional cardiologist assessed the coronary angiogram with an Optima IGS3 (General Electric, USA). Multiple projections on left and right coronary arteries were performed. A stenosis with a ≥ 70% reduction in the arterial lumen in ≥ 2 projections was considered significant for the three main epicardial arteries: left anterior descending artery (LAD), left circumflex artery (CX), right coronary artery (RCA), and eventually ramus intermedius (middle branch). A stenosis with a ≥ 50% reduction of the left main artery was considered significant. Patients with normal angiograms or non-significant stenosis were classified as the group CAD-, and those with significant stenosis as the group CAD+. Additionally, patients were classified according to the number of stenosed arteries and the severity of those stenoses by the SYNTAX score [13].

### Ethics

We obtained verbal consent from the patients after providing them with detailed information. Confidentiality and data security were ensured, and codes were used for patient identification.

### Statistical analysis

Statistical analyses were conducted using Statistical Package for Social Sciences SPSS 21 for Windows. Continuous variables are presented as mean ± standard deviation, while categorical variables are shown as percentages. The independent t-test was used for comparing means, ANOVA for multiple groups, and the chi-square test for comparing percentages. Pearson’s correlation coefficients assessed the relationship between continuous variables. A ROC curve was used to determine the cutoff value, sensitivity, and specificity of GLS values. To identify the ideal cut-off value, we used the Youden index. Multiple linear regression analysis was applied, and a p-value of less than 0.05 was considered significant. Interoperator reproducibility was calculated using the intraclass correlation coefficient.

## Results

### Characteristics of the study population

Fifty-two (52) individuals were finally included in our study. They were predominantly male (63.5%), with a mean age of 62.5 ± 11.9 years. All patients had a high pre-test probability of CCS according to the 2019-ESC-PTP model. They were divided into groups: group CAD- with non-significant CAD or normal arteries (48.1%, *n* = 25) and group CAD + with significant stenosis (51.9%, *n* = 27). The demographic, clinical, and echocardiographic characteristics of the two groups are summarized in Table 1. Hypertension, diabetes mellitus, dyslipidemia, and typical angina were significantly more frequent in the CAD + group. The pre-test probability was also significantly higher in the CAD + group (41.8 ± 16.7% vs. 28.6 ± 14.5%, *p* = 0.004). Clinical independent predictors of obstructive CAD presence were glycated hemoglobin (*p* = 0.04, OR = 2.38, 95 CI 1.04–5.46) and typical angina (*p* = 0.004, OR = 24.49, 95 CI 2.83-211.82).

**Table 1:** Demographic, clinical, and echocardiographic characteristics of the study population

Parameters	CAD- group (*n* = 25)		CAD + group (*n* = 27)	*p*
**Age (mean**,** years)**	59.9 ± 12.3	64.9 ± 11.2		0.133
**Sex**				0.618
Male, n(%)	15(60%)	18(66.7%)		
Female, n(%)	10(40%)	9(33.3%)		
**BMI ± SD (kg/m2)**	26.1 ± 3.0	25.5 ± 3.7		0.578
**Major CVRF**				
Age, n (%)	18(72%)	24(88,9%)		0.117
Sex, n (%)	7(28%)	8(29.6%)		0.897
Obesity, n (%)	3(12%)	2(7.4%)		0.462
Hypertension, n (%)	14(56%)	22(81.5%)		0.047
Diabetes mellitus, n (%)	9(36%)	19(70.4%)		0.013
Dyslipidemia, n(%)	15(60%)	23(85.2%)		0.041
Tobacco use, n (%)	0(0%)	3(33.3%)		0.231
Alcohol consumption, n (%)	0(0%)	2(7.4%)		0.265
**Chest pain**				
Typical angina, n (%)	7(28%)	19(70.4%)		0.002
Atypical angina, n (%)	16(64%)	6(22.2%)		0.002
Non anginal, n (%)	2(8%)	2(7.4%)		0.665
**CCS Angina Classification**				
Class 2, n (%)	23(100%)	20(80%)		0.031
Class 3, n (%)	0(0%)	5(20%)		
**Dyspnoea**,** n (%)**	14(56%)	15(55.6%)		0.974
**Pre-test probability ± SD (%)**	28.6 ± 14.5	41.8 ± 16.7		0.004
**Resting ECG**				
Abnormal T waves	15(60%)	19(70.4%)		0.432
**LVEF**,** mean ± SD (%)**	62.8 ± 6.2	62.7 ± 5.8		0.936
**E/e’**	8.6 ± 2.5	9.1 ± 2.1		0.440
**LA dilation**,** n (%)**	5 (20%)	5 (18.5%)		---
**GLS**,** mean ± SD (%)**	-18.99 ± 2.37	-15.89 ± 2.07		0.0001

BMI: Body Mass Index; CVRF: Cardiovascular Risk Factors; CCS: Canadian Cardiovascular Society; LVEF: Left Ventricular Ejection Fraction; PASP: Pulmonary Arterial Systolic Pressure; LAVI: Left Atrial Volume indexed; GLS: Global Longitudinal Strain

### GLS by 2D-STE diagnostic performance

The CAD + group significantly decreased GLS (-15.89 ± 2.07% vs. -18.99 ± 2.37% for the CAD- group, *p* = 0.0001).

**Fig. 2:**
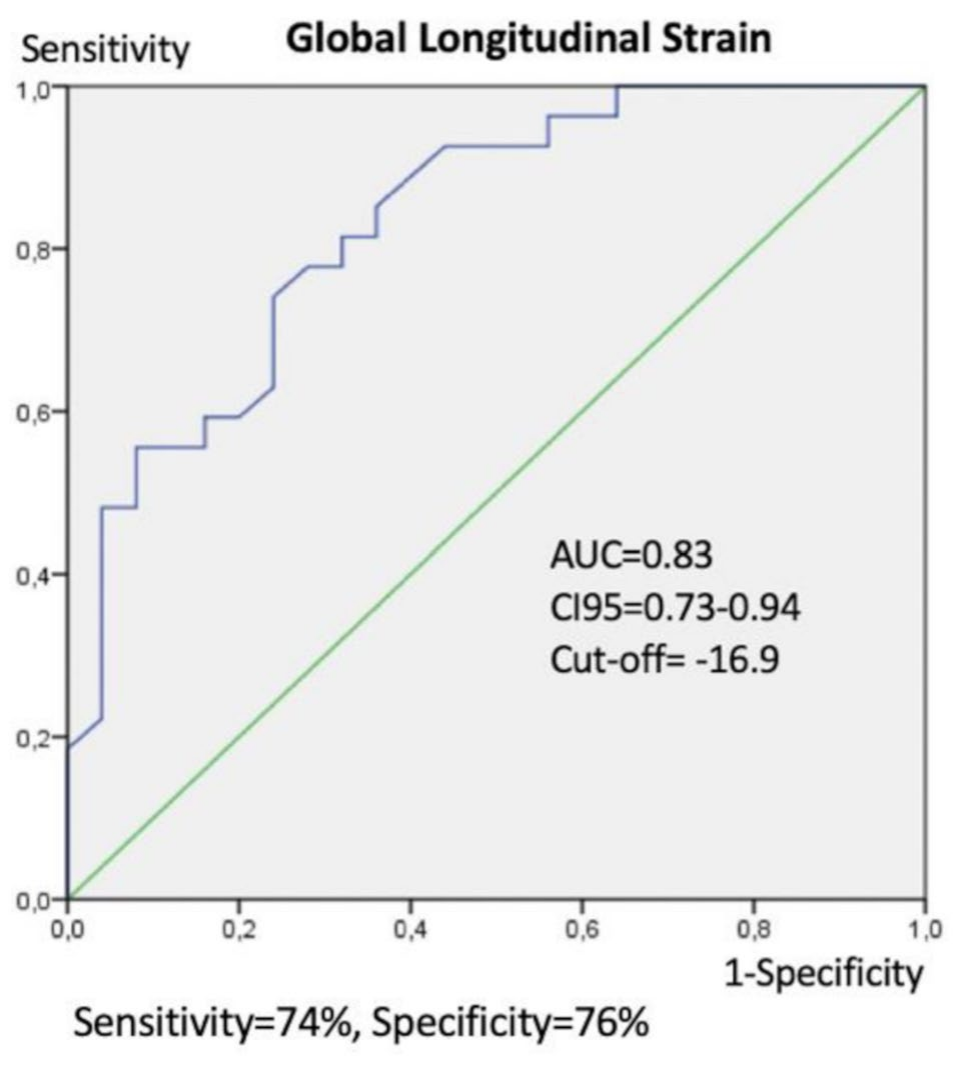
Receiver-operating characteristic (ROC) curve with area under the curve (AUC), 95% Confidence Interval (CI95) for AUC, and best cut-off value for strain when detecting the presence of significant CAD

The best cut-off value of 2D strain for detecting the presence of significant coronary lesions was − 16.9%, with a sensitivity of 74% and a specificity of 76%. The area under the curve (AUC) was 0.83, with a 95% confidence interval (CI) ranging from 0.73 to 0.94. (Fig. 2). The positive predictive value (PPV) was 76.9%, and the negative predictive value was 73.1%.

**Fig. 3:**
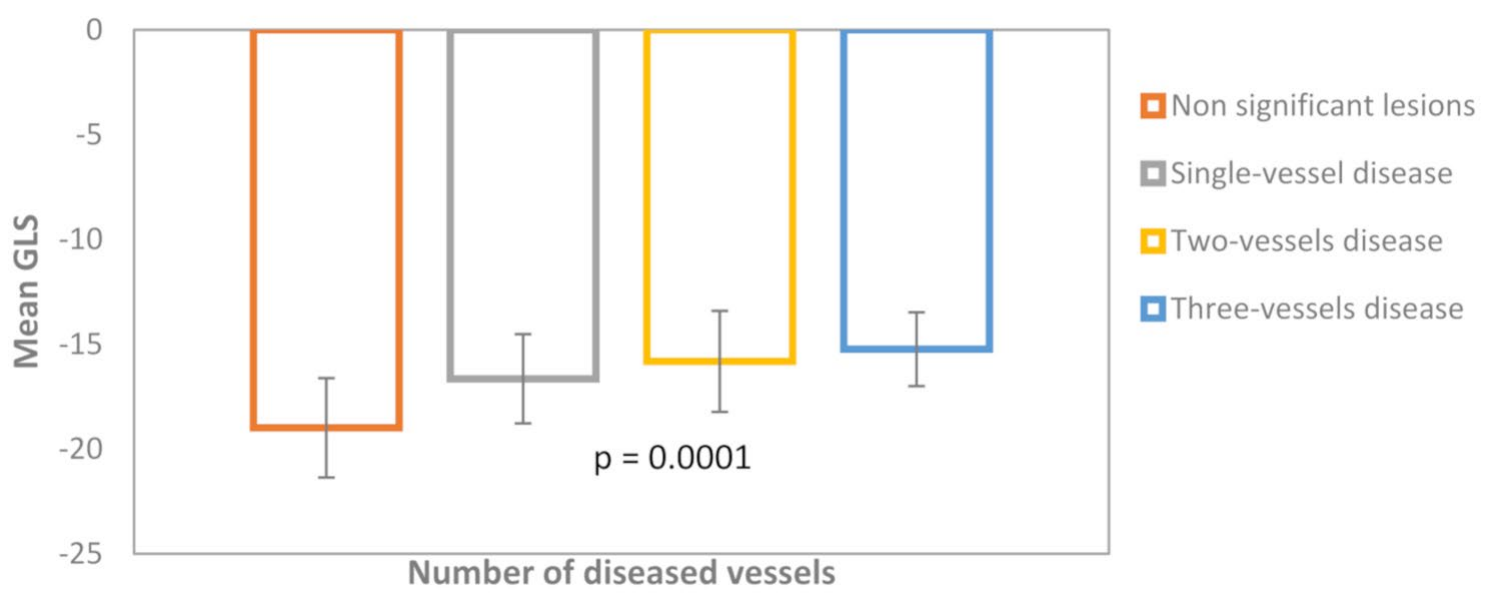
Mean GLS values and standard deviations of patients classified according to the number of diseased vessels

The GLS value was linearly correlated to the number of diseased vessels (*p* = 0.0001) (Fig. 3). There was no correlation between GLS and the complexity of coronary lesions assessed by SYNTAX score in the CAD + group (*p* = 0.18, *r* = 0.27). The multivariate analysis reveals that GLS was the only echocardiographic independent predictor factor of the presence of significant CAD at the angiogram (*p* = 0.002, OR = 2.07, 95%CI 1.30–3.30).

### Regional longitudinal strain

**Fig. 4:**
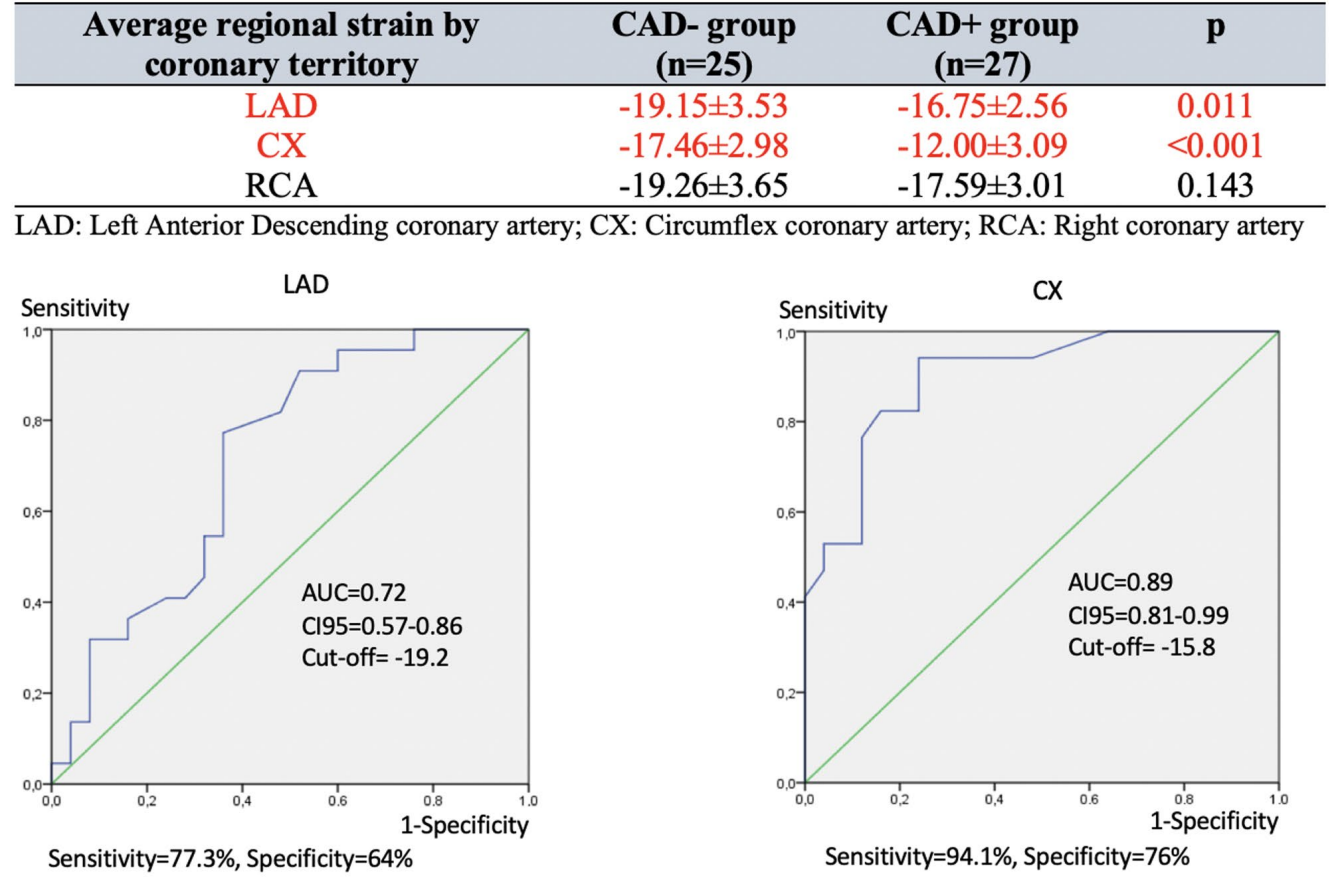
The two groups’ average regional strain by coronary territory (CAD- and CAD+). Receiver-operating characteristic (ROC) curves with respective area under the curve (AUC), 95% Confidence Interval (CI95) for AUC, and best cut-off value for strain when assessing coronary territory for Left Anterior Descending (LAD) and Circumflex (CX) coronary arteries

Regional strain is significantly reduced in patients with obstructive CAD in the left anterior descending coronary artery (LAD) (*p* = 0.01) and in the circumflex coronary artery (CX) (*p* < 0.001). The strain is reduced but non-significantly for patients with obstructive right coronary artery (RCA) (*p* = 0.143). Receiver-operator characteristic (ROC) curves denote a higher diagnostic best cut-off value for the LAD (-19.2%) compared to the CX (-15.8%). Figure 4 shows the average regional strain by coronary territory in the upper part and the ROC curves in the lower part.

### Reproducibility

GLS was performed with good reproducibility. The intraclass correlation coefficient (ICC) is 0.94 (95% IC 0.87–0.97, *p* < 0.0001).

## Discussion

### Pre-test probability

In our study, all patients had a high PTP score of CCS according to the 2019-ESC-PTP model, but only 51.9% of them had obstructive CAD confirmed by coronary angiogram, which suggests a high false-positive rate. The 2019 ESC PTP model could overestimate the likelihood of the disease in a substantial proportion of our population. The 2024 guidelines for the management of CCS underlined these issues. They proposed a new model, the Risk-Factor-weighted Clinical Likelihood model (RF-CL), which considers cardiovascular risk factors (CVRFs) and enhances the prediction performance of previous models [4]. Indeed, CVRFs were more frequent, and PTP scores were more elevated in the CAD + group in our study. Hence, using RF-CL could enhance the selection of patients who should undergo an angiogram.

On the other hand, these findings suggest that the PTP models might improve the prediction of significant CAD, but their accuracy may vary across different populations worldwide [14–16]. To our knowledge, these models have not been validated in sub-Saharan populations. In this context, using other non-invasive diagnostic tests like rest or stress echocardiography with GLS evaluation in high-probability patients could be beneficial in enhancing prediction performance before further invasive explorations [5, 6].

### GLS diagnostic performance

GLS significantly decreased in the CAD + group (-15.89 ± 2.07% vs. -18.99 ± 2.37% for the CAD- group, *p* = 0.0001). When comparing our results with those from other studies, the significance of the difference between CAD + and CAD- patients is constant. Table 2 compares studies on resting GLS to predict obstructive CAD. GLS has demonstrated good accuracy (AUC≥0.80), with acceptable to good sensitivity and specificity (> 70%) in our study and those of Bar et al. [11], Radwan et Hussein [17], Gaibazzi et al. [18] and Shimoni et al. [19]. A meta-analysis performed by Liou et al. showed similar results to ours with a GLS of -16.5% for CAD + patients vs. -19.7% for CAD- patients, an AUC of 0.81, a sensitivity of 74.4% and a specificity of 72.1% [8]. These results show that GLS is a highly sensitive and specific marker of the presence of obstructive CAD. However, the absence of a standardized best cut-off value for the detection of CAD is still a pending issue. The optimal cut-off values significantly vary across the studies due to methodological differences: the definition used of significant CAD (≥50% or 70% obstruction), the sample size, the selection of study populations (PTP model used, percentage of individuals with double or triple-vessel disease, the diastolic function at the moment of evaluation), and the 2D-STE software used for evaluation.

**Table 2:** Studies on resting LV GLS to predict obstructive CAD using coronary angiography as the reference standard

Studies, number of patients	CAD stenosis cutoff point	Mean GLS ± SD (%) in CAD + pts vs. CAD- pts, *p*-value	Best cut-off value (%)	Sensitivity/ Specificity (%)	PPV/ NPV (%)	AUC (95 CI)
Our study, *n* = 52	70%	−15.89 ± 2.07 vs. −18.99 ± 2.37, *p* = 0.0001	-16.9	74 / 76	76.9 / 73.1	0.83 (0.73–0.94)
Gaibazzi et al. [18], *n* = 82	50%	−19.02 ± 2.45 vs. − 22.73 ± 2.89, *p* < 0.001	−20.7	81.6 / 84.8	---	0.66
Bar et al. [11], *n* = 100	70%	−16.34 ± 1.48 vs. −20.76 ± 1.39, *p* < 0.001	−18.3	100 / 96.6	---	0.96 (0.99−1.00)
Biering Sorensen et al. [30], *n* = 293	70%	−17.1 ± 2.5 vs. −18.8 ± 2.6, *p* < 0.001			---	0.67 (−0.60–0.73)
Radwan et al. [17], *n* = 80	70%	−11.86 ± 2.89 vs. − 18.65 ± 0.79, *p* < 0.0001	−15.6	93.1 / 81.8	93.1 / 81.8	0.88 (0.78–0.96)
Shimoni et al. [19], *n* = 97	50%	−17.3 ± 2.4 vs. − 20.8 ± 2.3, *p* < 0.001	−19.7	81 / 67		0.80
Montgomery et al. [33], *n* = 123	50%	−16.8 ± 3.2 vs. − 19.1 ± 3.4, *p* = 0.0002	− 17.8		66 / 76	0.72 (0.63–0.82)

### GLS linearly correlated with the number of diseased vessels, not with the syntax score

As in many other studies, the number of diseased vessels was negatively correlated to the GLS in our study (*p* = 0.0001) [7, 11, 17, 18]. Therefore, GLS could be a valuable and cost-effective tool to evaluate the CAD severity and progression, refine risk stratification and prognosis [20–22], and guide treatment decisions (beta-blocker initiation, percutaneous intervention) [23, 24].

Our study did not find any correlation between GLS and SYNTAX Score (SS) (*p* = 0.17, *r* = 0.27), contrary to many other studies [25–28]. The SYNTAX score (SS) is an angiographic tool that evaluates CAD’s complexity and severity by considering not only the number of vessels but also the location of blockages, the degree of stenosis, and the presence of chronic total occlusions. This score gives a structural perspective on CAD to predict the optimal revascularization option (either percutaneous coronary intervention or coronary artery bypass grafting) [29]. GLS gives functional information related to the presence of myocardial damage secondary to coronary artery lesions but also to other factors such as microvascular obstruction, ischemia, and diastolic dysfunction. Therefore, a GLS can be moderately decreased or normal in a patient with high SS, depending on the extent of its compensating collateral circulation. Conversely, GLS can be impaired in a patient with low SS, in particular conditions (diabetes or hypertension), where the myocardial damage can be unrelated to obstructive CAD. These facts could explain the absence of a correlation between GLS and SS in our study, in which hypertension, diabetes, and dyslipidemia were significantly more prevalent in the CAD + group.

### Regional strain was reduced in patients with CAD in LAD and CX, not RCA

Some authors explored the possibility of using regional strain to predict the obstructed vessel [17, 30]. In our study, the diagnostic accuracy was excellent for the detection of CX stenosis but moderate for LAD stenosis, as for Zuo et al., who found an AUC of 0.818 for CX, 0.764 for LAD, and 0.723 for RCA in non-diabetic patients [31]. The same author found that the GLS might be less accurate in diagnosing obstructive CAD in diabetic patients than in non-diabetic patients [32]. These data suggest that, beyond the anatomical variability of coronary vascularization among individuals and the overlapping of the three epicardial artery territories, the development of collateral circulation and myocardial damage (unrelated to epicardial artery obstruction in specific diseases) could make it challenging to identify the culprit lesions through RLS only.

### Strengths and limitations

To our knowledge, this study is the first one conducted in sub-Saharan Africa, evaluating the diagnostic performance of GLS in high-probability patients to detect significant CAD. Hence, it enhances the external validity of the findings for practice in sub-Saharan Africa. GLS is a non-invasive, reproducible, accessible, and reliable tool, making it a very interesting option to consider when selecting patients for coronary angiography in regions with limited resources.

However, the small sample size and the single-center design limit the generalizability of our results. In addition, using the 2019 ESC PTP model (the validated tool at the beginning of our study) in patient selection may not reflect the specificity of these populations that are vastly underrepresented in studies. The last validated tool, RF-CL [4], which enhances the pre-test probability assessment by integrating classical risk factors, can be an alternative before establishing risk scores adapted to our context.

## Conclusion

Global longitudinal strain (GLS), assessed by 2D-STE at rest, is a strong predictor of the presence of significant coronary artery obstruction and the number of diseased vessels, with a good diagnostic performance in high clinical likelihood performance. As a cost-effective, reproducible, and non-invasive tool, it is a promising parameter that can improve the selection of patients undergoing coronary angiography in resource-limited settings.

### Recommendation

Further large studies are required in Africa, particularly in the validation of prediction models and diagnostic tools, in order to find the most beneficial interventions and the best patient profiles to reduce inadequate health expenditures.

## Data Availability

No datasets were generated or analysed during the current study.
